# Supplementary material for: Implementation strategies to increase human papillomavirus vaccination uptake for adolescent girls in sub-Saharan Africa: A scoping review protocol
Source: PLoS One. 2022 Aug 25;17(8):e0267617. doi: 10.1371/journal.pone.0267617 (PMC9409582; doi:10.1371/journal.pone.0267617)
Supplement: S2 Appendix — (DOCX) [file pone.0267617.s002.docx]

| First Author full names | Country of study | Year of publication | Title | Type of programme | Study design | Sample size | Targeted stakeholder | Frame work for data collection | Age of girls/school grade | Age of girls out of school | Data collecting tool | Implementation strategies | Funding source for the vaccine | HPV vaccine coverage/uptake |
| --- | --- | --- | --- | --- | --- | --- | --- | --- | --- | --- | --- | --- | --- | --- |

**SAMPLE DATA CHARTING FORM**
